# The Orthodontic Mini-Implants Failures Based on Patient Outcomes: Systematic Review

**DOI:** 10.1055/s-0043-1772249

**Published:** 2023-10-17

**Authors:** Siti Harlianti Putri Tarigan, Erliera Sufarnap, Siti Bahirrah

**Affiliations:** 1 Orthodontic Department, Faculty of Dentistry, Universitas Sumatera Utara, Medan, North Sumatera, Indonesia

**Keywords:** PROSPERO Registration: CRD42022337684 (16/06/22), mini-implant, miniscrew, temporary anchorage device, orthodontic anchorage, mini-implant failure

## Abstract

Anchorage is a challenge and essential issue for an orthodontist in determining the success of orthodontic treatment. Orthodontic anchorage is defined as resistance to unwanted tooth movement. Mini-implant is one of the devices that can be used as an anchor in orthodontic treatment. Many cases have reported successful treatment using mini-implant, but there are cases where mini-implants may fail. Failure of mini-implants can affect orthodontic treatment, and it is known that several factors may lead to mini-implant loss in orthodontic treatment. This systematic review aimed to determine the factors influencing mini-implant failure in orthodontic treatment. Articles were selected from electronic databases (PubMed, Google Scholar, The Cochrane Library, ScienceDirect) from January 2015 until 2023 according to the PRISMA method ( *Preferred Reporting Items for Systematic Reviews and Meta-Analysis* ) under the PEOS (Population-Exposure-Outcome-StudyType) framework questions for systematic review. The study was registered in the International Prospective Register of Systematic Reviews (PROSPERO) database (CRD42022337684). All data collected were in English, and filtering was done by eliminating duplicate data, meta-analysis, case reports, case series, mini-reviews, and animal studies. The analysis was further divided into three groups, that is, patient-related, implant-related, and operator-related and operator-related (A graphical abstract provided as a Supplementary information [available in the online version]). Twenty-one articles were identified according to the inclusion criteria in the form of retrospective, prospective, *in vivo* , and randomized controlled trial studies. Mini-implant failures due to patient-related showed six etiological factors, failures due to implant-related had eight etiological factors, and only one factor was operator-related, which may lead to mini-implant failure. The data was extracted without a computerized system and only in English. Mini-implant failure can be caused by many factors; we could not accuse one major factor as a cause. However, the quality or condition of the bones and oral hygiene are factors that play a significant role in obtaining the stability of implants. Mini-implant failure is highly influenced by poor oral hygiene and peri-implant inflammation. Comprehensive diagnostic prior to mini-implant insertion should be appropriately considered. This systematic review describes several factors that can influence mini-implant failure, divided into three groups: patient-related, implant-related, and operator-related (A graphical abstract provided as a Supplementary information [available in the online version]).

## Introduction

Anchorage is a challenge and important issue for an orthodontist in determining the success of orthodontic treatment. 1 There are two types of anchorage: extraoral (headgear or facemask) and intraoral anchorage (transpalatal arch or lingual arch). 2 Orthodontic anchorage is defined as resistance to unwanted tooth movement. 3 Several terms can be used to describe a mini-implant as an intraoral anchorage, such as miniscrew, miniscrew implant, microscrew, microscrew implant, and temporary anchorage devices (TADs). 3 4 5

Mini-implant material must have sufficient strength to prevent torque from the implant threads during insertion and removal without permanent deformation. The material must be nontoxic, biocompatible, and have good mechanical properties. 6 Safiya Sana and Huang et al divided implant material properties into biotolerant, bioinert, and bioactive. 6 7 Mini-implant insertion can be done by self-tapping (pre-drilling) and self-drilling methods. 8

The success rate of mini-implants has been widely reported to vary through previous studies from 74 to 93%. 9 10 11 According to some researchers, mini-implants are successful if the implants are stable in the jawbone until the end of treatment or until the planned time of removal. 12 13 Kaul and Dhanani mentioned that the mini-implants' success could be interpreted by the presence of minimal mobility and inflammation and the ability to obtain function correction through direct and indirect anchorage. 1 Similar to orthodontic mini-implant, according to Rodrigues et al, the quality and quantity of bones, such as their density and morphology, were the only predisposing factors for the successful osseointegration of dental implants. 14 Oral hygiene and peri-implant inflammation must be controlled to minimize the failure of implants. Bacterial infections are a common cause of endoosseous mini-implant failure. 15 Various studies have suggested reducing inflammation rates by taking daily mouthwash safely. 16 17 According to Nugraha et al, they concluded that *Robusta Green Coffee Bean* (RGCB) ethanol extract might be effective against periimplantitis bacteria *in vitro* , and chlorogenic acid in RGCB has antibacterial, anti-inflammatory, antioxidant, pro-osteogenic properties, and antibone resorption. 18

According to some researchers, mini-implant failure is interpreted as the occurrence of implant mobility less than 8 months after insertion, causing the implants to be unable to act as an anchor, and implant replacement is required. 10 19 20 Joshi et al said that there was a 30% increase in mini-implant failure due to peri-implant soft tissue inflammation. 21

Many factors cause mini-implant failure, so the possibility of failure must be considered. 22 The purpose of this systematic review is to provide information about the factors that may influence the occurrence of implant failure in orthodontic treatment.

## Methods

### Population-Exposure-Outcome-Study Type (PEOS) Question

This systematic review followed the Preferred Reporting Items for Systematic Reviews and Meta-Analysis (PRISMA) guidelines, and the study was registered in the PROSPERO database on 16/06/22 with the ID number CRD42022337684. The analysis was performed to answer the question “What causes orthodontic mini-implant failure?” according to:

**P** opulation: Patients in orthodontic treatment.
**E** xposure: The application of mini-implant.
**O** utcome: Mini-implant failure.
**S** tudy type: Randomized controlled trial, retrospective, prospective, *in vivo* .

**Table 1:** Inclusion and exclusion criteria of manuscripts

Inclusion Criteria
- Articles from databases PubMed/Google Scholar/ScienceDirect/The Cochrane Library. - Articles published from 2015-2023 - Articles' titles contain “orthodontic,” “mini-implant/mini-screw/micro implant/TAD/temporary anchorage device” - The article's title contains the word “failure” - Randomized controlled trial, retrospective, prospective, *in vivo* studies
**Exclusion Criteria**
- Dental implant - Animal studies - A systematic review, meta-analysis, case report, and case-series articles.

Abbreviation: TAD, temporary anchorage device.

### Information Sources and Literature Search

A systematic electronic search was limited to English language articles, and they were selected from electronic databases: PubMed, Google Scholar, ScienceDirect, and The Cochrane Library, published from January 2015 until 2023. The following keywords terms used for identification were “orthodontic” AND “mini-implant” OR “miniscrew” OR “micro implant” OR “temporary anchorage device” OR “TAD” AND “failure” OR “fail.” The keywords were adjusted related to orthodontic mini-implant with the title “failure” and manually searched for relevance to the content.

### Study Selection

The study was limited to research articles or original articles, that is, case–control trials, cohort studies, retrospective studies, case series, randomized controlled trials, or cross-sectional trials. Articles were then removed to Mendeley Library. Duplicates, systematic reviews, meta-analyses, case reports, and case-series articles were removed. Two authors independently searched and screened for the articles based on the search database engine for the titles and the abstracts. Exclusion criteria were adjusted to search for the eligible articles. Information and articles were checked, approved, and reviewed by the authors (an orthodontic resident with two supervisors until all disagreements had been solved to achieve the final results. Twenty-one articles fulfilled the inclusion criteria.

### Data Collection, Measurements, and Risk of Individual Bias Analysis

**Fig. 1:**
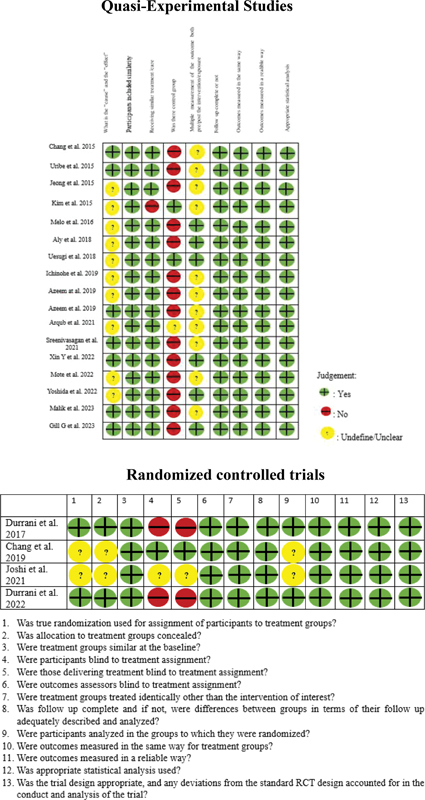
Risk of bias assessment for the studies included in this review.

The data extraction was performed individually by two reviewers. The data extracted was based on the inclusion and exclusion criteria ( Table 1 ): Study characteristics (manuscript in English, authors name, authors country, year of publication, and study design), sample characteristics (grouping: implant placement site, sample size, mini-implant size, gender and age of participant), outcome assessment (evaluation of failure criteria), and result. The risk of bias or critical appraisal of each study was analyzed by two reviewers who individually used Joanna Briggs Institute (JBI), quasi-experimental studies, or nonrandomized controlled trials, and the risk of bias for randomized controlled trials studies form presented in Fig. 1 . 23 24

## Results

### Studies Included Characteristics

Articles were selected according to the inclusion and exclusion criteria from the various databases. The studies obtained 32.576 articles from all databases; after screening and eligibility, authors found 73 papers were accepted for the full-text assessment. It was selected according to the criteria and duplication checks, so 39 articles were obtained. The 39 articles included six systematic reviews and meta-analyses, one case report, two studies that used machines or models, two book-compiled studies, three finite element analyses, one in Portuguese language, and two literature reviews. Seventeen of these articles were removed, and a further one was removed due to an insufficient result from the JBI's critical appraisal and was inconsistent with the topic. Twenty-one articles were concluded to be selected that matched the inclusion criteria.

**Fig. 2:**
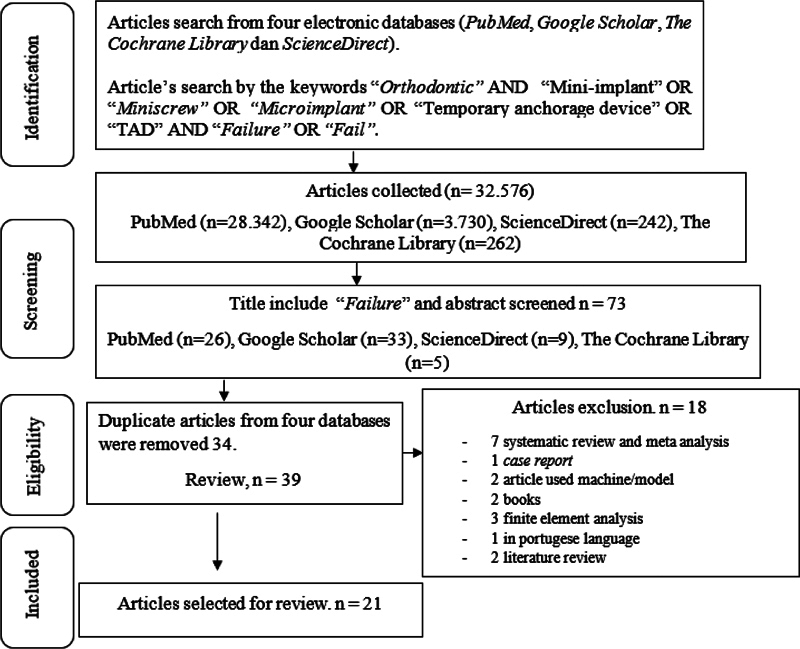
Pathway of Preferred Reporting Items for Systematic Reviews and Meta-Analyses (PRISMA) flow diagram for study selection.

Reviewers decided to have a minimum mean from two reviewers of 70% of JBI's critical appraisal answered with yes to each article to achieve the standard quality of the article. Among the twenty-one analyzed studies, thirteen articles had above 80%, 8 articles had below 80% with appropriate answers. The characteristic diagram workflow that fulfilled the criteria is presented in Fig. 2 and Table 2 .

**Table 2:** Characteristics of included articles systematic review

No	Author/year/country/study	Mini-implant samples/ failure	Mini-implant			Gender Samples	Age (years) Sample	Failure criteria/time	Result
			Size (mm) Samples	Site	Force load				
1	• Chang et al, ^65^ • 2015 • Taiwan • *Retrospective analysis*	1680/7.2%	2 × 12	mandibular buccal shelf (moveable mucous vs. attached gingiva)	227-397g/ Immediate loading (power chain)	♀:435♂:405	16 ± 5	Undefined/ 3.3 months	• Patient-related: age 14 ± 3 years, placement side • Implant-related: - • Operator-related: left-sided implant
2	• Uribe et al, ^19^ • 2015 • USA • *Retrospective cohort study*	55/21.8%	2 × 9	Infrazygomatic	200g	♀:42 ♂:13	22 ± 11	Mobility and loose/ undefined	• Patient-related: poor oral hygiene, medical condition, ♂ > ♀ • Implant-related: failure diameter/length of 1.5-1.8mm/6-8mm • Operator-related: force > 150g, left-sided implant, inexperienced operator
3	• Jeong et al, ^73^ • 2015 • Korea • Undefined	331/ 17.22%	1.2 × 7	Buccal alveolar bone maxillae/ mandible	1,4,8,12,16 weeks loading	Undefined	20.08 ± 7.52	Mobility/1-5 weeks	• Patient-related: • Implant-related: force • Operator-related: failure in the first week of force loading
4	• Kim et al, ^13^ • 2016 • Korea • Undefined	394/18.27%	1.2 × 7	Buccal alveolar bone	Undefined	♀:101 ♂:24	21.2 ± 7.6	Mobility and loose	• Patient-related: ♂ > ♀, mandibula > maxillae, young > adult patient • Implant-related: • Operator-related:-
5	• Melo et al, ^9^ • 2016 • Brazil • *Retrospective (cross-sectional study)*	1356/10.9%	1.3, 1.4, 1.6 x 5,7,9,11	Buccal, lingual, palatal or alveolar crest	Immediate loading	♀:423 ♂:147	21.95 ± 7.6	Mobility and fractured	• Patient-related: male > female, mandibula > maxillae, palatal site > other site. • Implant-related: Arch, smaller size > bigger size, &design • Operator-related:-
6	• Durrani et al, ^72^ • 2017 • Pakistan • *Randomized controlled trial, in vivo*	60/ Single thread: 13,3% Dual thread:20%	Total length: 13 2 × 10	Maxillary arches between the roots of the second premolar and the first molar	300g/Immediate loading (nickel-titanium coil spring	30 ♀:67%	Mean: 18 Range: 14-20	Undefined	• Patient-related: • Implant-related: arch, thread design • Operator-related: failure in the first month after insertion
7	• Aly et al, ^22^ • 2018 • Egypt • *Prospective clinical trial*	180/17,8%	1.5, 1.6, 1.8 x 6, 8, 10	Buccal, palatal/left, right/maxilla and mandible	50g, 100g, 150g, 200g, 250g	♀:58 ♂:24	21.41	Sudden spontaneous loss or the presence of mobility or looseness during routine visits that required replacing the TAD used or infected painful pathological condition that could be seen as normal inflammation	Patient-related: age, oral hygiene, gender, frequency of brushing per day Implant-related: type, size • Operator-related:loading
8	• Uesugi et al, ^71^ • 2018 • Japan • *Retrospective study*	387/20,9%	1.4, 1.6 (maxillary buccal), 1.6, 2.0 x 6, 8 (midpalatal)	Maxillary buccal, midpalatal suture	Undefined	♀:176 ♂:62	27.9 ± 8.4	Mobility, inflammation, and anchorage function last less than one year after orthodontic loading	Patient-related: Implant-related: size • Operator-related:
9	• Ichinohe et al, ^70^ • 2019 • Japan • Undefined	25/20% Cortical bone thickness (<1.5 mm), 32% Screw-suture distance (<1.5 mm), 20% insertion depth (<4.5 mm)	2 × 9	Median palate	Dynamic loads 2-4N	♀:18 ♂:7	23.4 ± 5.6 Range: 15.0–34.5	Natural loss or mobility	Patient-related: bone condition Implant-related: Operator-related:
10	• Chang et al, ^61^ • 2019 • Indianapolis (USA) • *Randomized double-blind clinical trial*	772/6.3%	2 × 1	Infrazygomatic crest	397g or 389 cN/ Immediate loading	♀:310 ♂:76	Mean age: 24.3 10.3–59.4	Loose (mobile) screw that exfoliated or was deemed too loose to provide effective anchorage	Patient-related: age Implant-related: material, arch Operator-related:
11	• Azeem et al, ^12^ · 2019 · Pakistan · *Retrospective pilot study*	60/26,3%	1.3, 1.5 × 8, 10	Maxillary tuberosity region	Immediate loading 100–150g (elastomeric or nickel-titanium coil springs 12 mm)	♀:23 ♂:17	20.1 ± 8.9	Mobility or loose during orthodontic treatment	Patient-related: oral hygiene, placement side Implant-related: Operator-related: experience
12	• Azeem et al, ^69^ · 2019 · Pakistan · *Retrospective cohort study*	110/23,2%	1.3, 2 x 8, 10	Buccal retromolar area, at the distobuccal surface of the second molars, is between the anterior border of the mandibular ramus and the temporal crest.	Immediate loading 100–150g (elastomeric chain or nickel-titanium coil springs)	♀:52 ♂:55	Mean age: 18.6 SD: 5.2	Mobility or loosened	Patient-related: oral hygiene, placement side Implant-related: location Operator-related:
13	• Arqub et al, ^34^ • 2021 • USA • *Retrospective cohort study*	275/ Palatal: 8.5% Buccal: 32.5%	2 x 8, 10	Palatal and buccal	Undefined	127	–	Undefined	Patient-related: gender, malocclusion type Implant-related: location, the treatment objective Operator-related:
14	• Joshi et al, ^21^ • 2021 • India • *Randomized controlled trials and cross-sectional*	Undefined	1.4 × 8	Between the second premolar and the first molar of the maxilla	Immediate loading 150g (power chain or power chain with SS ligature wire)	30	15-30	Inflammation	Patient-related: Implant-related: auxiliary attachment Operator-related:
15	• Sreenivasagan et al *,* ^30^ • 2021 • India • Undefined	218/16,5%	Undefined	Anterior interradicular, buccal shelf, infrazygomatic, palatal and midline	Undefined	♀: 142 ♂: 76	15.5 ± 8.3	Complications included breaking, soft tissue coverage needing excision, poor oral hygiene and tissue overgrowth	Patient-related: oral hygiene Implant-related: insertion location Operator-related:
16	• Xin et al *,* ^36^ • 2022 • China • Retrospective	889/17.10% (failed once), 5.29% (failed twice or more)	6.0 × 1.4 8.0 × 1.4 10.0 × 2.0	Retromaxillary and retromandibular area	50–200 g	♀: 292 ♂: 55	25.62 ± 7.43	Mobility and cannot accomplished their clinical purpose	Patient-related: age Implant-related: insertion location Operator-related: -
17	• Mote et al *,* ^51^ • 2022 • India • Descriptive study	195/61.67%	-	Maxilla (attached and unattached gingiva)	Undefined	−	20-45	Undefined	Patient-related:- Implant-relatd: insertion location Operator-related:-
18	• Yoshida et al *,* ^35^ • 2022 • Japan • Retrospective study	46715.6%	1.3 to 1.6 mm and 5.0 to 8.0 mm	Attached gingiva on the buccal side	Delayed loading	♀: 156 ♂: 41	Mean age: 20 years and 2 months (range: 12 years 5 months–47 years 1 month)	Movement of the implant	Patient-related: gender, age Implant-related: dental arch Operator-related: -
19	• Durrani ^54^ • 2023 • Pakistan • Randomized clinical control trial split-mouth design	184/ HA-coated (11%), uncoated group (13%)	1.6 × 8 The pitch of the threads was 0.8 mm throughout the length of the TAD	At the mucogingival junction at angulation 45° to the long axis of the tooth	Directly loaded 300 g with a nickel-titanium coil spring	92	–	Loosening the TAD to a degree in which it could not sustain the force of the coil spring	Patient-related: - Implant-related: location insertion Operator-related: -
20	• Malik et al *,* ^25^ • 2023 • Pakistan • Retrospective review	85/± 26%	8 × 1.5 -	Maxilla and mandibula	Undefined	♀: 46 ♂: 39	Mean age: 20 years	Not stable with inflammation or any pathological condition around the implant	Patient-related: bone condition and gender Implant-related: - Operator-related: -
21	• Gill et al *,* ^42^ • 2023 • India • Prospective study	64/28.1%	12/14 × 2	Infrazygomatic crest	Immediate and after 2 weeks	32	18–33 with an average age of 25 years	Loss of the miniscrew in less than 8 months after placement	Patient-related: oral hygiene and placement side Implant-related: force load Operator-related: -

## Discussion

Anchorage is essential in orthodontics; its control is needed for the best treatment result. 25 Orthodontic mini-implants are routinely used worldwide as orthodontic anchorage because they can be easily placed and do not require patient compliance. 26 Although some studies reported the success rate of mini-implants between 80 and 100%, mini-implant failure might occur frequently, 27 as some studies reported the failure rate could be between 5 and 20%. 22 25 While dental implants require osseointegration for stability, retention for orthodontic implant does not involve osseointegration but facilitated by mechanical interlocking at the implant-bone interface. 28

Mini-implant is supposed to be stable, and no need to replace it until the end of the orthodontic treatment. If the implant showed signs of looseness, inflammation, or mobility, so it needs to be removed and replaced, the implant would be considered failed. 29 According to Sreenivasagan et al, mini-implant failure can be considered when there is excessive implant movement, soft tissue coverage, or loosening. 30 Orthodontic mini-implants failure can be influenced by several factors, which will be classified in this review into: patient-related, implant-related, and operator-related.

They were related to the patient, including gender, age, oral hygiene, bone condition, type of malocclusion, and placement side. Based on the mini-implant, including insertion location, dental arch, treatment objective, size, design, force, material, auxiliary attachment, and the operator related to the experience. Four studies reported that implant failure is more common in men, whereas according to a study conducted by Baik et al 31 and Rasool et al *,* no difference was found in the success rate between men and women. 32 According to Maki et al *,* men also exhibit higher bone density than women, which can differ from the oral cavity site. 33 The higher failure rate in men may be related to anatomic and hormonal differences 34 and the presence of thicker and denser cortical bone, leading to the application of excess torque during implantation. 35

The patient's age also affects the stability of the implants. Xin et al stated that implants inserted in older patients tended to be more stable than in younger patients. 36 Lee et al found that younger patients have lower bone density and finer cortical bone 37 this is also similar to that reported by Fayed et al 38 and Farnsworth et al, 39 whereas Chen et al 40 stated that bone density is higher in older age patient and this was supported by Präger et al 41 who found that older patients have greater cortical thickness and are more likely to have more excellent stability.

Several studies reported a higher failure rate in patients with poor oral hygiene. 12 22 30 42 Oral hygiene is known to be a local risk factor for mini-implant failure since mini-implant stability depends on adequate oral hygiene. 43 44 Similar to the studies in this review, Sharma et al 45 also reported that mini-implant loss related to poor oral hygiene and local inflammation; meanwhile according to Park et al 46 ^,^ oral hygiene played no role, but local inflammation around the mini-implants does. Inflammation damages the bone around the neck of the bone screws; progressive damage to the cortical bone causes mobility and exfoliation. 44

The oral cavity is a unique microenvironment that contains different types of microbes. 47 Study conducted by Zhao et al to analyze the contribution of the oral microbiome to the failure of TAD reported that the failed group showed enriched pathogenic genes involved in oxidative phosphorylation, flagellar assembly, and bacterial chemotaxis. There is no osseointegration in the TAD insertion site. Therefore, microorganisms inducing TAD failure might not be analogous to peri-implantitis. 48 Peri-implantitis is a polymicrobial disease caused by plaque accumulation and retention. 49 50 In the Zhao et al study, they found gram-negative species that could induce host inflammation, such as *Eikenella corrodens, Neisseria elongata, Prevotella intermedia, and Citronella morbi* around failed TAD. 48

Bone condition or quality is a critical factor affecting mini-implants' stability. 51 Kim et al stated, according to Misch, the maxillary alveolar bone is mainly composed of porous bone, corresponding to D3 or D4; meanwhile, the mandible has dense bone classified as D2 and D3. 52 The maxillary buccal cortical bone between the first molar and second premolar is thickest and the anterior area tends to have denser bone than posterior area. 51 Ntolou et al stated that sites with thin attached gingiva (AG), thick cortical bone, plenty of available bone, and dense cancellous bone are ideal for mini-implant placement, since they increase the chances of preventing local inflammation, achieving proper primary stability, and also achieving and maintaining secondary stability. 53

The higher failure rate occurs more frequently in the mandible than the maxilla. 42 54 Five studies in this review showed the failure rate higher in mandible as well as Miyawaki et al, 43 Park et al, 46 and Wiechmann et al, 55 all found implants to be more successful in the maxilla, as maxilla has greater amount of keratinized tissue, less demanding surgical procedures, and greater vascularization as compared to the mandible. Similar to the others, Papageorgiou et al also reported that significantly higher failure rate occurs in the mandible (28.2%) compared to the maxilla (11.8%). 20 This is may be related to the variations in bone structure such as mineral density and alveolar cortical bone thickness. 56 57

The optimal onset of force application has long been a disputed issue as to whether a healing period is necessary for mini-implants stability. Previous research reported immediate loading might destabilize implants and result in more failures. 58 According to Chen et al, inflammation control and delayed loading were still necessary for mini-implants to achieve sufficient primary stability even after 3 weeks of healing, although osseointegration was not required at this stage. 59 Gill et al 42 concluded that loading of the mini-implant should be done after a latent period of 2 weeks. However, Nkenke et al found no differences in daily bone apposition, bone-implant contact, and bone density in the presence or absence of early loading. 60

Chang et al reported stainless steel (SS) mini-implants had an insignificantly higher failure rate than titanium alloy implants. 61 Various failure rates have been reported; to reduce the failures, there are various surface treatments of the implant's exterior have been recommended. 62 63 Recently, active surfaces of prosthetic implants have been introduced with antimicrobial, growth factors, or hydroxyapatite (HA) on the implant surface. 64 However, study conducted by Durrani to compare the stability of HA-coated with uncoated TAD concluded that TADs coated with HA do not have any statistical difference in the failure when placed on the buccal shelf of the maxilla so the premise that the HA-coated TADs will have a lower failure rate seems incorrect. 54 The failure rate also higher when inexperienced operators placed implants so it is suggested that the insertion should be carried out by an experienced person or a resident supervised by a supervisor. 12 The detailed of this review can be seen below.

Orthodontic mini-implants failure can be influenced by several factors 8 , which will be classified into: patient-related, implant-related, and operator-related.

### Patient-Related

#### Gender

The result from Arqub et al studies to compare survival rates of palatal and buccal mini-implants and to evaluate risk factors that influenced the survival of mini-implants showed that the survival rate of buccal mini-implants in men is lower than in women (68.4 and 80.2%). This may be related to anatomic and hormonal differences. 34

Meanwhile, the result of Kim et al study to evaluate the failure rate of orthodontic mini-implants in the implant failure rate (IFR) and patient failure rate (PFR) showed that IFR and PFR were higher in men than women, although the difference was not significant. 13 Similar to the others, Yoshida et al 35 and Malik et al 25 study also showed that the failure rates of implant were significantly higher in males compared to females. According to Ono et al, the cortical bone between first molar and the second premolar is often the site of implantation thicker in men than in women. 56

#### Age

Aly et al conducted a prospective clinical trial on 82 patients with a mean age of 21.41 years and showed a greater failure rate at less than or equal to 20 years than at more than 20 years. 22 Similar to Aly et al, the results of Chang et al study on 840 patients with mandibular buccal shelf miniscrews showed that failure occurred in patients with a mean age of 14 ± 3 years, which was below the overall mean age (16 ± 5 years). 65

Xin et al 36 conclude that younger people with removable appliances that implant inserted in the retromaxillary or retromandibular regions had a higher progressive susceptibility to loosening, while the result of Yoshida et al 35 study showed that the failure rate was highest among patients aged more than or equal to 30 years compared to less than 20 years group.

Some studies reported that a greater failure rate generally occurs at a younger age because they have less dense cortical bone, where bone quality is a major factor in the success of orthodontic mini-implants. 22 66 67 Meanwhile, Du et al examined age-related changes in the mandible found that volumetric bone mineral density increased with age in the 20 to 29, 30 to 39, and 40 to 49 years age groups but decreased in the 50 years age group. 68

#### Oral Hygiene

Mini-implant failure can occur due to poor oral hygiene. The results of Azeem et al, 12 Aly et al, 22 Uribe et al, 19 Azeem et al, 69 Sreenivasagan et al, 30 and Gill et al 42 showed a greater failure rate in patients with poor oral conditions.

Oral hygiene is a significant factor in the success rate of mini-implants since the stability of the mini-implant depends on adequate oral hygiene. Poor oral hygiene can cause food accumulation and inflammation around the mini-implant that can lead to failure, while good oral hygiene can reduce inflammation around the mini-implant. 22 34 42

#### Bone Condition

Ichinohe et al conducted a study to determine the stability of mini-implants placed in the median palate area, which showed that the success rate of mini-implants was significantly higher in patients with an implant-suture distance of 1.5 to 2.7 mm than 0 to 1.4 mm. The mini-implant can also be more stable with a palatal cortical bone thickness greater than 1.5 mm and an insertion depth of more than or equal to 4.5 mm. 70

Malik et al study conclude that bone quality is a significant factor that impacts the clinical performance of orthodontic mini-implant. 25 According to Ntolou et al, sites with dense cancellous bone, plenty of available bone, thin AG, and thick cortical bone are ideal for mini-implant insertion since that increase the chances of achieving proper primary and secondary stability and prevent local inflammation and also the cancellous bone and thin cortical bone with very low density adversely affect the success of mini-implant. 53

#### Type of Malocclusion

Arqub et al showed that mini-implants in class III malocclusion patients had a lower success rate than class II malocclusion. This was related to the type of mechanism used in the mini-implant. Generally, in class II patients, a mini-implant was used for retracting anterior maxillary teeth, while in class III, implants are typically placed in the buccal shelf or retromolar pad for distalization of the entire mandibular arch. 34

#### Placement Side

The result of Azeem et al and Azeem et al showed a higher failure rate of mini-implant insertion on the right side. 12 69 This was associated with better oral hygiene on the left side in right-handed patients. Good oral hygiene can reduce inflammation around the mini-implants. 65

Meanwhile, Gill et al showed a higher failure rate for infra zygomatic implants placed on the left side (31.3%) than on the right side (25.0%). This indicates the technical sense of the possibility and procedure of other uncontrolled biological factors such as unilateral preference for mastication unequal level of oral hygiene among left and right-handed patients. 42

### Implant-Related

#### Insertion Location

Arqub et al study on 127 patients with a total of 275 mini-implants in the palatal and buccal areas showed that there was no significant difference between maxillary and mandibular buccal implants, although the survival rate of buccal mini-implants was lower in the mandible. Comparison of the mini-implants survival rate in buccal alveolar, infra zygomatic, buccal shelf, and palatal areas showed a significant difference with the lowest survival rate in the buccal shelf area. 34

Azeem et al study showed a 23.2% failure rate of mini-implants in the retromolar area due to inflammation around the mini-implant, so to minimize mini-implant failure in the retromolar area, a clinician should try to reduce inflammation around the mini-implants. 69 According to Mote et al study, the success rate of mini-implant in maxillary arch at AG was higher than at unattached gingiva 80 and 61.67%, respectively. This result may be due to the risk of fracture and failure during insertion increase as the mini-implant diameter decreases. 51

Xin et al reported that the retromaxillary and retromandibular areas were the worst places for insertion, whereas the palatal area was considered to be the ideal placement sited because of the lower possibility of contacting the root. 36 Durrani et al study aims to compare the stability of HA-coated with uncoated implants and concluded that implants coated with HA do not have any statistical difference in the failure when placed on the buccal shelf of the maxilla. 54

#### Dental Arch

Kim et al compared PFR and IFR in the maxilla and mandible and then found a higher failure rate in the mandible than the maxilla though not significant. 13 Aly et al 22 and Melo et al 9 said that the loss of stability of mini-implants was more significant in the mandible than in the maxilla. Sreenivasagan et al also reported that mobility leading to failure occurs more often on the right side and in the mandible than the maxilla. 30

The higher failure rate is due to the mandible having thicker cortical bone, which can cause the bone to overheat during drilling, higher insertion torque, short AG zone causing difficult insertion in the AG, and narrower vestibular area preventing the patient from being able to clean the mandibular area thoroughly. 9 22

Similar to other studies, Yoshida et al also reported that the failure rates significantly higher in the mandible than maxilla. This may be related to variations in bone structure such as alveolar cortical bone thickness and mineral density. 35

#### Treatment Objective

The biomechanical objectives of the mini-implants have a significant effect on the palatal mini-implant success rate. The results of Arqub et al study showed that there was a substantial difference in the mini-implant success rate based on treatment needs. The lowest success rate occurred in the palatal mini-implants used for distalization (70.1%). This is influenced by the quality and quantity of bone and the influence of various host factors and oral hygiene, which can reduce the success rate. 34

#### Size and Design

Melo et al used mini-implants with a length of (5, 7, 9, and 11 mm) and a diameter of 1.3, 1.4, and 1.6 mm). There was no significant difference based on the mini-implant diameter, but the mini-implant with a shorter length (5 mm) showed a higher failure rate. 9 Aly et al study showed no difference related to the type and length of the mini-implant, although mini-implants with a length of 6 mm had the lowest success rate compared to 8 and 10 mm. The success rate was lowest for mini-implants with a diameter of 1.5 mm than 1.6 and 1.8 mm. 22

Uesugi et al concluded that a miniscrew with a length of 8 mm was significantly more stable than 6 mm for primary and secondary insertions in the molar buccal area. As an orthodontic anchorage, there is no osseointegration between the mini-implant and the bone, so mini-implant stability is provided by the mechanical interdigitation of the implant and surrounding bone. Therefore, implants with a longer size will be more stable because the area of interdigitation with bone is larger. 71

Durrani et al compared single-thread and dual-thread implants made of *titanium-aluminum-vanadium alloy* with the same dimensions (total length: 13 mm, thread length: 10 mm, and diameter: 2 mm). The result of a clinical trial on 60 implants followed for a minimum of 10 months and a maximum of 18 months showed that four implants failed in the single thread group and six implants failed in the dual thread group but showed no significant relationship between thread design and mini-implant failure. Dual-thread mini-implant exhibits better mechanical properties due to greater maximum insertion torque, greater maximum disengagement torque, and greater pullout force than single-thread mini-implants. 72

#### Force

Azeem et al applied a direct force ( *immediate loading* ) of 100 to 150 g using a elastomeric or nickel-titanium coil spring (12 mm) and showed a greater failure rate occurred in mini-implants with a direct force of more than 100 g using elastomeric, although the difference was not significant. 12

Aly et al found that applying force directly to the mini-implant after insertion resulted in a higher success rate than delayed loading. Immediate loading after insertion is a safe technique, with a higher success rate than delayed force, and can accept loads of up to 250 g. 22

However, Jeong et al concluded that 75% of mini-implant failures occur within 16 weeks after insertion. A high failure rate occurs when the force is applied after fewer than 12 weeks of insertion. Good stability of mini-implants is obtained about 3 to 4 months after insertion. The highest failure rate according to the time of force after insertion occurred when the mini-implant was loaded during the first week after insertion. They concluded that immediate loading after insertion could activate bone resorption and lead to the failure of the mini-implants. Failure after applying force is often observed for up to 13 weeks. Therefore, it is necessary to follow up on the mini-implants stability 3 months after the loaded force. 73

Gill et al study compared implant failure with immediate and delayed time till loading showed a 100% success rate in delayed loaded implant group (after 2 weeks) compared to loaded immediately (60.9%). 42 According to Papageorgiou et al *,* in most cases, premature loading leads to healing by forming fibrous tissue between the bone and the mini-implant. 20

#### Material

Chang et al compared SS and titanium alloy (TiA) mini-implant failure rates placed in the infra zygomatic crest area of the AG and moveable mucosa (MM) and concluded that SS mini-implants had an insignificantly higher failure rate than TiA and a higher failure rate occurred in SS mini-implant at AG than TiA. 61

#### Auxiliary Attachment

Several auxiliary attachments can be used for space closure, such as Ni-Ti closing spring, elastomeric power chain, and either direct or indirect use of SS ligature wire. Joshi et al study to evaluate mini-implant failure during retraction used an elastomeric chain and SS ligature wire. Failure evaluation was marked with gingival inflammation around the mini-implant and showed that a power chain is more likely to cause gingival inflammation than SS-ligature that is attached to the power chain. 21

### Operator-Related

Several studies compared experienced and inexperienced operators performing mini-implant placement; it was seen that the failure rate was higher when implants were placed by inexperienced operators. As the results of Azeem et al study to evaluate the failure rate of mini-implants placed in the maxillary tuberosity area showed a significant relationship to the mini-implants placement by inexperienced operators, then to minimize mini-implant failures it is suggested by an experienced operator. 12

We acknowledge the potential limitations as a part of this systematic review. The manuscripts were limited in an English language only, and some manuscripts written in languages other than English may lead to the language bias. The limitations of this review can be overcome by using a computerized system to filter data using applications that support systematic reviews making, whereas for the selected language, author can only choose the articles in English due to the inability to understand other foreign languages.

## Conclusion

Although orthodontic mini-implant success is reported to be high, implant failure in orthodontic treatment is possible. Many factors and interrelated can cause mini-implants loss. This systematic review describes several factors that can influence mini-implant failure, divided into three groups: patient-related, implant-related, and operator-related. The quality or condition of the bones and oral hygiene are factors that play a crucial role in obtaining the stability of implants. Mini-implant failure is highly influenced by poor oral hygiene and peri-implant inflammation. Three main factors that caused failures were described from the clinical point of view research and further assessment should be considered as comprehensive matters that could involve a mechanical simulation or *in vivo* research.

## References

[1] Kaul Y Dhanani D CA Temporary anchorage devices Int Healthc Res J. 2017 1 03 6 11

[2] Kuroda S Tanaka E Risks and complications of miniscrew anchorage in clinical orthodontics Jpn Dent Sci Rev 2014 50 04 79 85

[3] Elias C N de Oliveira Ruellas A C Fernandes D J Orthodontic implants: concepts for the orthodontic practitioner Int J Dent 2012 2012 549761 23209470 10.1155/2012/549761 PMC3502859

[4] Papadopoulos M A Tarawneh F The use of miniscrew implants for temporary skeletal anchorage in orthodontics: a comprehensive review Oral Surg Oral Med Oral Pathol Oral Radiol Endod 2007 103 05 e6 e15 10.1016/j.tripleo.2006.11.022 17317235

[5] Morais L S Serra G G Muller C A Titanium alloy mini-implants for orthodontic anchorage: immediate loading and metal ion release Acta Biomater 2007 3 03 331 339 17257912 10.1016/j.actbio.2006.10.010

[6] Safiya Sana S S Mini-implant materials: an overview IOSR J Dent Med Sci 2013 7 02 15 20

[7] Huang L H Shotwell J L Wang H L Dental implants for orthodontic anchorage Am J Orthod Dentofacial Orthop 2005 127 06 713 722 15953897 10.1016/j.ajodo.2004.02.019

[8] Singh K Kumar D Jaiswal R K Bansal A Temporary anchorage devices - mini-implants Natl J Maxillofac Surg 2010 1 01 30 34 22442547 10.4103/0975-5950.69160 PMC3304189

[9] Melo A CM Andrighetto A R Hirt S D Bongiolo A LM Silva S U Silva M A Risk factors associated with the failure of miniscrews - a ten-year cross sectional study Braz Oral Res 2016 30 01 e124 27783770 10.1590/1807-3107BOR-2016.vol30.0124

[10] Antoszewska J Papadopoulos M A Park H S Ludwig B Five-year experience with orthodontic miniscrew implants: a retrospective investigation of factors influencing success rates Am J Orthod Dentofacial Orthop 2009 136 02 1580 1.58E12, discussion 158–159 10.1016/j.ajodo.2009.03.031 19651342

[11] Sarul M Minch L Park H S Antoszewska-Smith J Effect of the length of orthodontic mini-screw implants on their long-term stability: a prospective study Angle Orthod 2015 85 01 33 38 24745630 10.2319/112113-857.1 PMC8634807

[12] Azeem M Haq A U Awaisi Z H Saleem M M Tahir M W Liaquat A Failure rates of miniscrews inserted in the maxillary tuberosity Dental Press J Orthod 2019 24 05 46 51 10.1590/2177-6709.24.5.046-051.oar PMC6833927 31721946

[13] Kim J-W Lee N-K Sim H-Y Yun P-Y Lee J-H Failure of orthodontic mini-implants by patient age, sex, and arch; number of primary insertions; and frequency of reinsertions after failure: an analysis of the implant failure rate and patient failure rate Int J Periodont Restor Dent 2016 36 04 559 565 10.11607/prd.2675 27333014

[14] Rodrigues J MM Santos P L Mendonça G Faloni A PS Finoti L S Margonar R Assessment of deviations of implants installed with prototyped surgical guide and conventional guide: in vitro study Eur J Dent 2023 17 01 39 45 36063845 10.1055/s-0040-1718791 PMC9949936

[15] Hussain M W Abullais S S Naqash T A Bhat M YS Microbial etiology and antimicrobial therapy of peri-implantitis: a comprehensive review Open Dent J 2019 12 01 1113 1122

[16] Utami W S Anggani H S Purbiati M Cytotoxicity effect of orthodontic miniscrew-implant in different types of mouthwash: an in-vitro study J Orthod Sci 2022 11 01 5 35282292 10.4103/jos.jos_158_21 PMC8895380

[17] Cortizo M C Oberti T G Cortizo M S Cortizo A M Fernández Lorenzo de Mele M A Chlorhexidine delivery system from titanium/polybenzyl acrylate coating: evaluation of cytotoxicity and early bacterial adhesion J Dent 2012 40 04 329 337 22305778 10.1016/j.jdent.2012.01.008

[18] Nugraha A P Ardani I GGW Sitalaksmi R M Anti-peri-implantitis bacteria's ability of Robusta green coffee bean (Coffea Canephora) ethanol extract: an in silico and in vitro study Eur J Dent 2023 17 03 649 662 36075265 10.1055/s-0042-1750803 PMC10569850

[19] Uribe F Mehr R Mathur A Janakiraman N Allareddy V Failure rates of mini-implants placed in the infrazygomatic region Prog Orthod 2015 16 01 31 26373730 10.1186/s40510-015-0100-2 PMC4571029

[20] Papageorgiou S N Zogakis I P Papadopoulos M A Failure rates and associated risk factors of orthodontic miniscrew implants: a meta-analysis Am J Orthod Dentofacial Orthop 2012 142 05 577 5.95E9 23116500 10.1016/j.ajodo.2012.05.016

[21] Joshi R Shyagali T R Jha R Gupta A Tiwari A Tiwari T Evaluation and comparison of the effect of elastomeric chain and stainless steel ligature wire on maxillary orthodontic miniscrew failure Int J Appl Basic Med Res 2021 11 02 100 105 33912430 10.4103/ijabmr.IJABMR_191_20 PMC8061618

[22] Aly S A Alyan D Fayed M S Alhammadi M S Mostafa Y A Success rates and factors associated with failure of temporary anchorage devices: a prospective clinical trial J Investig Clin Dent 2018 9 03 e12331 10.1111/jicd.12331 29512336

[23] Munn Z Barker T H Moola S Methodological quality of case series studies: an introduction to the JBI critical appraisal tool JBI Evid Synth 2020 18 10 2127 2133 33038125 10.11124/JBISRIR-D-19-00099

[24] Barker T H Stone J C Sears K The revised JBI critical appraisal tool for the assessment of risk of bias for randomized controlled trials JBI Evid Synth 2023 21 03 494 506 36727247 10.11124/JBIES-22-00430

[25] Malik F Khan F Ali S Rana F Haq H Hussain M Factors affecting success and failure of orthodontic mini-implants: a retrospective review Prof Med J 2023 30 02 285 291

[26] Ikenaka R Koizumi S Otsuka T Yamaguchi T Effects of root contact length on the failure rate of anchor screw J Oral Sci 2022 64 03 232 235 35644561 10.2334/josnusd.21-0536

[27] Schätzle M Männchen R Zwahlen M Lang N P Survival and failure rates of orthodontic temporary anchorage devices: a systematic review Clin Oral Implants Res 2009 20 12 1351 1359 19793320 10.1111/j.1600-0501.2009.01754.x

[28] Crismani A G Bertl M H Čelar A G Bantleon H P Burstone C J Miniscrews in orthodontic treatment: review and analysis of published clinical trials Am J Orthod Dentofacial Orthop 2010 137 01 108 113 20122438 10.1016/j.ajodo.2008.01.027

[29] Dalessandri D Salgarello S Dalessandri M Determinants for success rates of temporary anchorage devices in orthodontics: a meta-analysis (n > 50) Eur J Orthod 2014 36 03 303 313 23873818 10.1093/ejo/cjt049

[30] Sreenivasagan S Subramanian A K Rengalakshmi S Prevalence and cause of mini-implant failure encountered by orthodontic residents J Long Term Eff Med Implants 2021 31 04 1 4 10.1615/JLongTermEffMedImplants.2021035979 34587408

[31] Baik U-B Bayome M Han K-H Park J H Jung M-H Kook Y-A Evaluation of factors affecting the success rate of orthodontic mini-implants by survival analysis World J Stomatol. 2013 2 03 56 61

[32] Rasool G Mahmood Shah A Rahman S Hussain U Saeed A Gul P Success rate of different insertion sites and lengths of success rate of different insertion sites and lengths of mini screws in orthodontic patients JKCD 2018 8 01 49 53

[33] Maki K Miller A Okano T Shibasaki Y Changes in cortical bone mineralization in the developing mandible: a three-dimensional quantitative computed tomography study J Bone Miner Res 2000 15 04 700 709 10780862 10.1359/jbmr.2000.15.4.700

[34] Arqub S A Gandhi V Mehta S Palo L Upadhyay M Yadav S Survival estimates and risk factors for failure of palatal and buccal mini-implants Angle Orthod 2021 91 06 756 763 34003884 10.2319/090720-777.1 PMC8549566

[35] Yoshida M Nakano H Haga S Arishima T Maki K Investigation of the association between vertical skeletal patterns and the timing of failure of temporary anchorage devices Showa Univ J Med Sci 2022 34 04 191 199

[36] Xin Y Wu Y Chen C Wang C Zhao L Miniscrews for orthodontic anchorage: analysis of risk factors correlated with the progressive susceptibility to failure Am J Orthod Dentofacial Orthop 2022 162 04 e192 e202 35987884 10.1016/j.ajodo.2022.07.013

[37] Lee S J Ahn S J Lee J W Kim S H Kim T W Survival analysis of orthodontic mini-implants Am J Orthod Dentofacial Orthop 2010 137 02 194 199 20152674 10.1016/j.ajodo.2008.03.031

[38] Fayed M M Pazera P Katsaros C Optimal sites for orthodontic mini-implant placement assessed by cone beam computed tomography Angle Orthod 2010 80 05 939 951 20578867 10.2319/121009-709.1 PMC8939012

[39] Farnsworth D Rossouw P E Ceen R F Buschang P H Cortical bone thickness at common miniscrew implant placement sites Am J Orthod Dentofacial Orthop 2011 139 04 495 503 21457860 10.1016/j.ajodo.2009.03.057

[40] Chen Y J Chang H H Huang C Y Hung H C Lai E HH Yao C CJ A retrospective analysis of the failure rate of three different orthodontic skeletal anchorage systems Clin Oral Implants Res 2007 18 06 768 775 17868386 10.1111/j.1600-0501.2007.01405.x

[41] Präger T M Brochhagen H G Mischkowski R Jost-Brinkmann P G Müller-Hartwich R Bone condition of the maxillary zygomatic process prior to orthodontic anchorage plate fixation J Orofac Orthop 2015 76 01 3 13 25420944 10.1007/s00056-014-0261-5

[42] Gill G Shashidhar K Kuttappa M N Kushalappa P B D Sivamurthy G Mallick S Failure rates and factors associated with infrazygomatic crestal orthodontic implants - a prospective study J Oral Biol Craniofac Res 2023 13 02 283 289 36880016 10.1016/j.jobcr.2023.02.010 PMC9984842

[43] Miyawaki S Koyama I Inoue M Mishima K Sugahara T Takano-Yamamoto T Factors associated with the stability of titanium screws placed in the posterior region for orthodontic anchorage Am J Orthod Dentofacial Orthop 2003 124 04 373 378 14560266 10.1016/s0889-5406(03)00565-1

[44] Wu T Y Kuang S H Wu C H Factors associated with the stability of mini-implants for orthodontic anchorage: a study of 414 samples in Taiwan J Oral Maxillofac Surg 2009 67 08 1595 1599 19615569 10.1016/j.joms.2009.04.015

[45] Sharma P Valiathan A Sivakumar A Success rate of microimplants in a university orthodontic clinic ISRN Surg 2011 2011 982671 22084789 10.5402/2011/982671 PMC3195314

[46] Park H S Jeong S H Kwon O W Factors affecting the clinical success of screw implants used as orthodontic anchorage Am J Orthod Dentofacial Orthop 2006 130 01 18 25 16849067 10.1016/j.ajodo.2004.11.032

[47] Rosier B T Marsh P D Mira A Resilience of the oral microbiota in health: mechanisms that prevent dysbiosis J Dent Res 2018 97 04 371 380 29195050 10.1177/0022034517742139

[48] Zhao N Zhang Q Guo Y Oral microbiome contributes to the failure of orthodontic temporary anchorage devices (TADs) BMC Oral Health 2023 23 01 22 36650527 10.1186/s12903-023-02715-7 PMC9844000

[49] Belibasakis G N Manoil D Microbial community-driven etiopathogenesis of peri-implantitis J Dent Res 2021 100 01 21 28 32783779 10.1177/0022034520949851 PMC7754824

[50] Zhang Y Li Y Yang Y Periodontal and peri-implant microbiome dysbiosis is associated with alterations in the microbial community structure and local stability Front Microbiol 2022 12 785191 35145492 10.3389/fmicb.2021.785191 PMC8821947

[51] Mote N Rajbhar J Toshniwal N G Rathod R To evaluate success and failure rate of temporary anchorage devices (TADS) in various attachments sites in maxilla abstract Egypt Orthod J 2022 61 06 60 71

[52] Kim H J Yun H S Park H D Kim D H Park Y C Soft-tissue and cortical-bone thickness at orthodontic implant sites Am J Orthod Dentofacial Orthop 2006 130 02 177 182 16905061 10.1016/j.ajodo.2004.12.024

[53] Ntolou P Tagkli A Pepelassi E Factors related to the clinical application of orthodontic mini-implants J Int Oral Health 2018 10 03 103 110

[54] Durrani O K Comparison of in vivo failure of precipitation-coated hydroxyapatite temporary anchorage devices with that of uncoated temporary anchorage devices over 18 months Am J Orthod Dentofacial Orthop 2023 163 04 520 525 36503860 10.1016/j.ajodo.2022.03.015

[55] Wiechmann D Meyer U Büchter A Success rate of mini- and micro-implants used for orthodontic anchorage: a prospective clinical study Clin Oral Implants Res 2007 18 02 263 267 17348892 10.1111/j.1600-0501.2006.01325.x

[56] Ono A Motoyoshi M Shimizu N Cortical bone thickness in the buccal posterior region for orthodontic mini-implants Int J Oral Maxillofac Surg 2008 37 04 334 340 18295454 10.1016/j.ijom.2008.01.005

[57] Baumgaertel S Hans M G Buccal cortical bone thickness for mini-implant placement Am J Orthod Dentofacial Orthop 2009 136 02 230 235 19651353 10.1016/j.ajodo.2007.10.045

[58] Trisi P Rebaudi A Peri-implant bone reaction to immediate, early, and delayed orthodontic loading in humans Int J Periodontics Restorative Dent 2005 25 04 317 329 16089040

[59] Chen Y J Chang H H Lin H Y Lai E HH Hung H C Yao C CJ Stability of miniplates and miniscrews used for orthodontic anchorage: experience with 492 temporary anchorage devices Clin Oral Implants Res 2008 19 11 1188 1196 18983323 10.1111/j.1600-0501.2008.01571.x

[60] Nkenke E Lehner B Weinzierl K Bone contact, growth, and density around immediately loaded implants in the mandible of mini pigs Clin Oral Implants Res 2003 14 03 312 321 12755781 10.1034/j.1600-0501.2003.120906.x

[61] Chang C H Lin J S Roberts W E Failure rates for stainless steel versus titanium alloy infrazygomatic crest bone screws: a single-center, randomized double-blind clinical trial Angle Orthod 2019 89 01 40 46 30372127 10.2319/012518-70.1 PMC8137128

[62] Farronato D Mangano F Briguglio F Iorio-Siciliano V Riccitiello F Guarnieri R Influence of Laser-Lok surface on immediate functional loading of implants in single-tooth replacement: a 2-year prospective clinical study Int J Periodont Restor Dent 2014 34 01 79 89 10.11607/prd.1747 24396842

[63] Guarnieri R Grande M Ippoliti S Iorio-Siciliano V Riccitiello F Farronato D Influence of a laser-Lok surface on immediate functional loading of implants in single-tooth replacement: three-year results of a prospective randomized clinical study on soft tissue response and esthetics Int J Periodont Restor Dent 2015 35 06 865 875 10.11607/prd.2273 26509991

[64] Kim J E Kang S S Choi K H The effect of anodized implants coated with combined rhBMP-2 and recombinant human vascular endothelial growth factors on vertical bone regeneration in the marginal portion of the peri-implant Oral Surg Oral Med Oral Pathol Oral Radiol 2013 115 06 e24 e31 23706924 10.1016/j.oooo.2011.10.040

[65] Chang C Liu S SY Roberts W E Primary failure rate for 1680 extra-alveolar mandibular buccal shelf mini-screws placed in movable mucosa or attached gingiva Angle Orthod 2015 85 06 905 910 25603272 10.2319/092714.695.1 PMC8612035

[66] Kingsmill V J Boyde A Variation in the apparent density of human mandibular bone with age and dental status J Anat 1998 192(Pt 2):233 244 9643424 10.1046/j.1469-7580.1998.19220233.x PMC1467757

[67] Han S Bayome M Lee J Lee Y J Song H H Kook Y A Evaluation of palatal bone density in adults and adolescents for application of skeletal anchorage devices Angle Orthod 2012 82 04 625 631 22077190 10.2319/071311-445.1 PMC8845559

[68] Du X Jiao J Cheng X Age-related changes of bone mineral density in mandible by quantitative computed tomography J Biol Regul Homeost Agents 2017 31 04 997 1003 29254305

[69] Azeem M Saleem M M Liaquat A Ul Haq A Ul Hamid W Masood M Failure rates of mini-implants inserted in the retromolar area Int Orthod 2019 17 01 53 59 30770333 10.1016/j.ortho.2019.01.006

[70] Ichinohe M Motoyoshi M Inaba M Risk factors for failure of orthodontic mini-screws placed in the median palate J Oral Sci 2019 61 01 13 18 30369558 10.2334/josnusd.17-0377

[71] Uesugi S Kokai S Kanno Z Ono T Stability of secondarily inserted orthodontic miniscrews after failure of the primary insertion for maxillary anchorage: maxillary buccal area vs midpalatal suture area Am J Orthod Dentofacial Orthop 2018 153 01 54 60 29287652 10.1016/j.ajodo.2017.05.024

[72] Durrani O K Shaheed S Khan A Bashir U Comparison of in-vivo failure of single-thread and dual-thread temporary anchorage devices over 18 months: a split-mouth randomized controlled trial Am J Orthod Dentofacial Orthop 2017 152 04 451 457 28962727 10.1016/j.ajodo.2017.05.019

[73] Jeong J-W Kim J-W Lee N-K Kim Y-K Lee J-H Kim T-W Analysis of time to failure of orthodontic mini-implants after insertion or loading J Korean Assoc Oral Maxillofac Surg 2015 41 05 240 245 26568925 10.5125/jkaoms.2015.41.5.240 PMC4641214

