# Supplementary figures and images for: The Orthodontic Mini-Implants Failures Based on Patient Outcomes: Systematic Review
Source: Eur J Dent. 2023 Oct 17;18(2):417–29. doi: 10.1055/s-0043-1772249 (PMC11132791; doi:10.1055/s-0043-1772249)

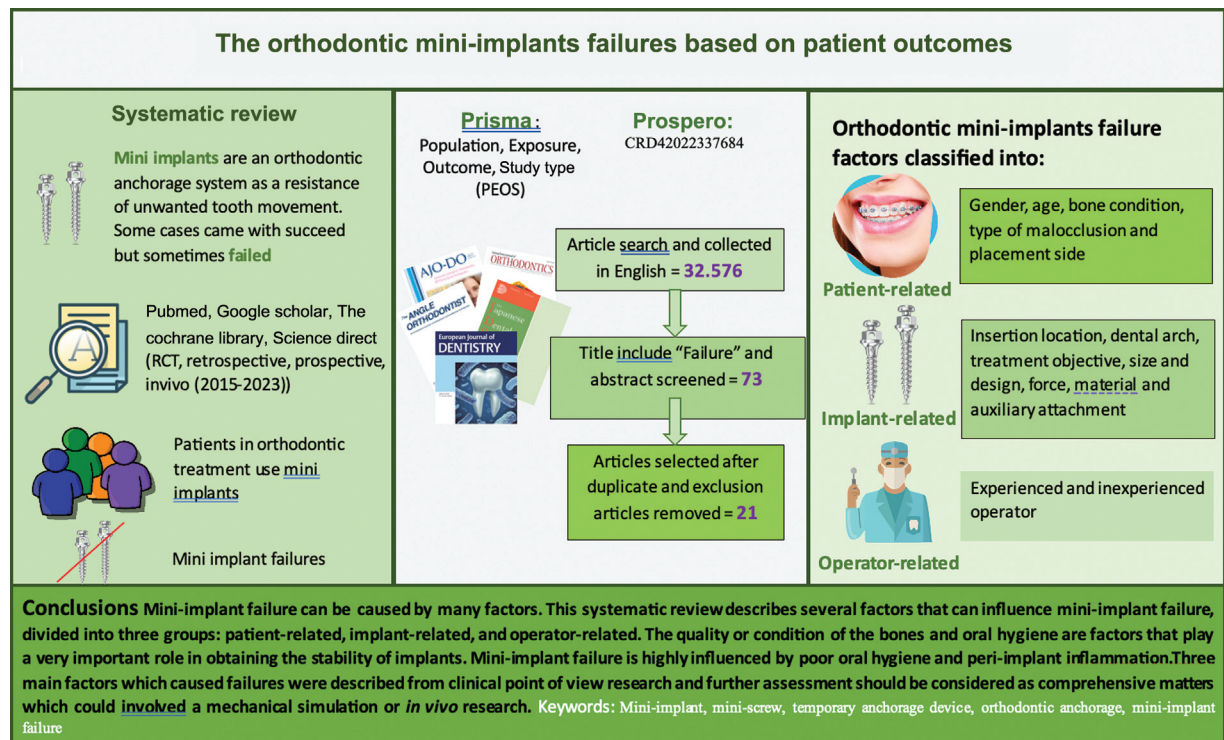

Supplement: Supplementary file 1 — Supplementary Material [file 10-1055-s-0043-1772249-s22122529.pdf]
